# Fulminant Infective Endocarditis Due to *Kingella Kingae* and Several Complications in a 6-Year-Old Girl: A Case Report

**DOI:** 10.3389/fped.2021.707760

**Published:** 2021-07-05

**Authors:** Raphael Joye, Dimitri Ceroni, Maurice Beghetti, Yacine Aggoun, Tornike Sologashvili

**Affiliations:** ^1^Pediatric Cardiology Unit, Geneva University Hospitals, Geneva, Switzerland; ^2^Pediatric Orthopedic and Traumatology Unit, Geneva University Hospitals, Geneva, Switzerland; ^3^Pediatric Cardiac Surgery Unit, Geneva University Hospitals, Geneva, Switzerland

**Keywords:** endocarditis, *Kingella kingae*, ischemic stroke, cardiac surgery, child

## Abstract

*Kingella kingae* is a gram-negative coccobacillus belonging to the HACEK group (*Haemophilus* species, *Aggregatibacter actinomycetemcomitans, Cardiobacterium hominis, Eikenella corrodens*, and *Kingella* species) and is a common oropharyngeal colonizer of healthy young children. Osteoarticular infection is the most commonly reported invasive *Kingella kingae* infection in children, usually presenting a mild clinical picture. However, it can also cause severe invasive infections, especially infective endocarditis, with a high complication rate. We report the case of a 6-year-old girl, with no past medical history, who presented with fulminant infective endocarditis due to *Kingella kingae*. She received emergency venoarterial extracorporeal membrane oxygenation support, rapidly underwent cardiac surgery, and was then treated using ceftriaxone for 4 weeks as recommended by the American Heart Association. The patient's postoperative course was marked by a cerebral ischemic stroke consistent with septic embolism. She also presented with a para-aortic pseudoaneurysm that required a secondary surgical procedure, with a good postoperative result. This report illustrates a case of fulminant infective endocarditis due to *Kingella kingae* and responsible for two major complications. We also describe the preventive valve surgery performed to ensure the preservation of valve function and its capacity for growth.

## Introduction

*Kingella kingae* (*K. kingae*) is a beta-hemolytic encapsulated coccobacillus belonging to the *Neisseriaceae* family of gram-negative bacteria often found colonizing the oropharynx of healthy young children (1–3). The colonized oropharyngeal mucosa thus acts as a gateway for *K. kingae* into the bloodstream, which disseminates it to distant sites. Bone and joint infections, including spondylodiscitis, are the most reported invasive *K. kingae* infections in children and usually present a milder clinical picture than those caused by other typical pathogens (4, 5). However, soft tissue infections, occult bacteremia, infective endocarditis (IE), and, more rarely, lower respiratory tract, meningeal, and ocular infections have also been reported in children, usually among those aged younger than 4 years old (6, 7).

IE refers to an infection of the endocardium and usually one or more heart valves. Recently, the epidemiology seems to have been evolving, with a higher incidence of IE in children, especially among those with congenital heart disease (8). Stockheim et al. reported that only 12% of cases occurred in children with a normal heart structure (9). The clinical presentation is often subacute, with prolonged low-grade fever and fatigue. However, a small proportion of cases evolves swiftly, with rapid deterioration. The most frequently encountered organisms in children are *streptococci* (mostly the *viridans* group), *staphylococci* (*S. aureus* and coagulase-negative *staphylococci*) (10). Most fulminant forms are caused by *staphylococci* (11). *K. kingae* is a rare cause of IE in children, predominantly before 3 years of age, but with high morbidity and mortality rates. Complications involving the central nervous system, such as septic embolisms, are the most commonly encountered in children with IE due to *K. kingae*, with a reported rate ranging from 20 to 30% (12, 13).

We report the case of a 6-year-old girl with endocarditis due to *K. kingae*, who required emergency venoarterial extracorporeal membrane oxygenation (ECMO) and surgery, with subsequent central nervous system complications and a para-aortic pseudoaneurysm. The patient's parents provided written informed consent for this report.

## Case Description

**Figure 1:**
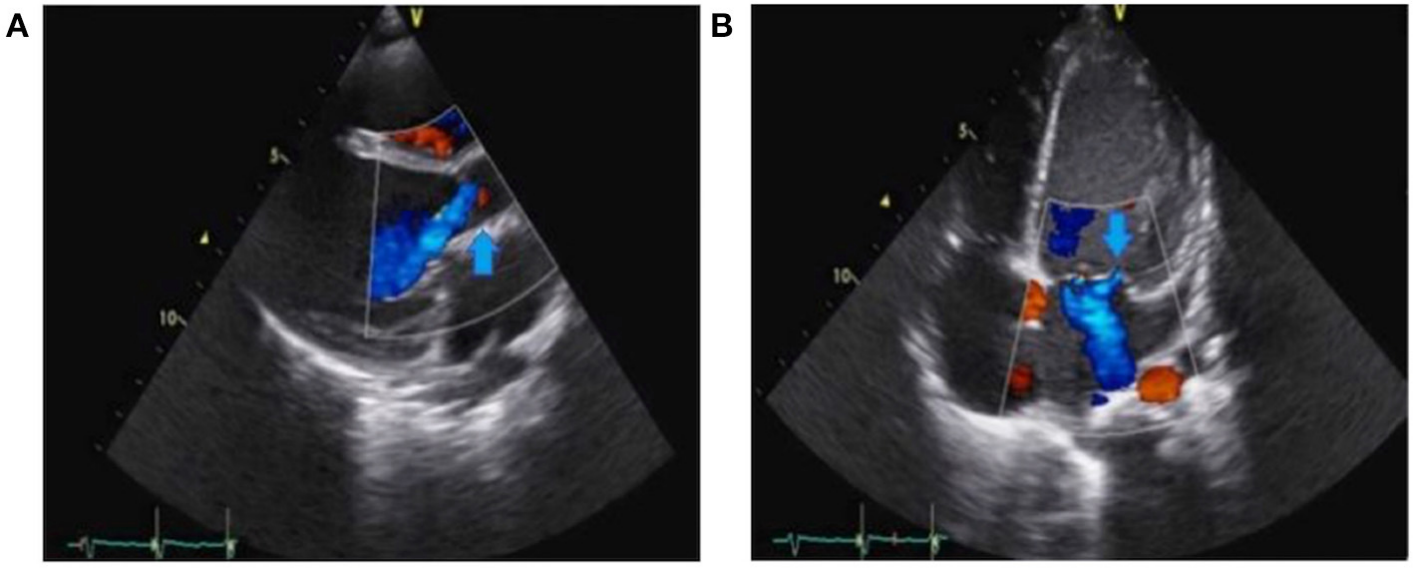
Preoperative transthoracic echocardiography (TTE) showing severe aortic regurgitation (arrow) in **(A)** and severe mitral regurgitation (arrow) in **(B)**.

**Figure 2:**
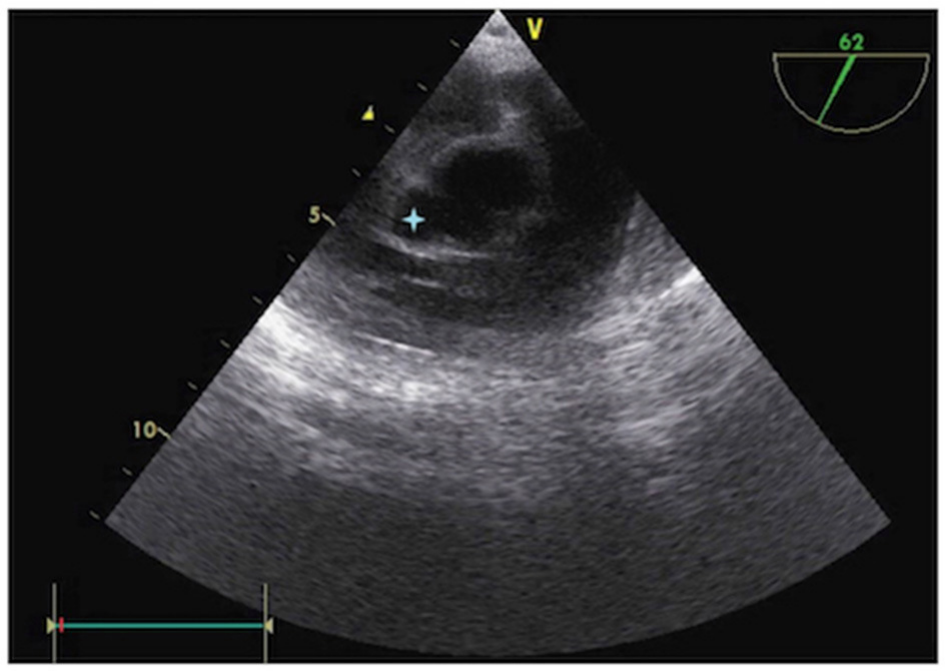
Preoperative transesophageal echocardiography (TEE) showing a para-aortic abscess (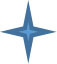).

A 6-year-old girl with no previous medical history presented with a 7-day history of poor general condition, fever of up to 40°C, and progressive lethargy arising in the context of oral aphthous stomatitis. She also complained of intermittent left precordial chest pain. Her parents were administering paracetamol 15 mg/kg per dose q.i.d. and ibuprofen 10 mg/kg per dose t.i.d. to treat fever and pain. An initial physical examination revealed no fever but measured tachycardia >150 beats/min associated with hypotension measured to 73/43 mmHg. Heart auscultation revealed muffled heart sounds and a diastolic murmur at the right upper sternal border. Clinical examination also highlighted hepatomegaly. No focal neurological abnormalities were noted. White blood cell count was elevated, at 25 × 10^3^/microliter, whereas C-reactive protein was 164 mg/L (normal < 10 mg/L). Troponin and the pro-brain natriuretic peptide (pro-BNP) were both extremely elevated at 658 ng/l (*N* < 14 ng/l) and > 70,000 ng/l (*N* < 300 ng/l), respectively. The electrocardiogram demonstrated diffuse depression of the ST segment and marked cardiomegaly was found on the thoracic radiograph. Transthoracic echocardiography (TTE) showed severe aortic and mitral regurgitations, with subsequent left chamber dilation causing severe left-ventricle systolic dysfunction (shortening fraction of 18%). Aortic valve vegetation was noted, and a para-aortic abscess was also suspected (Figure 1). The patient experienced rapid hemodynamic deterioration until a cardiac arrest required cardiac support using venoarterial ECMO. Transesophageal echocardiography confirmed the previous findings (Figure 2). A turbid liquid was collected at the opening of the pericardium and sent for culture and PCR investigations. A type 1 R-N bicuspid aortic valve, according to the Sievers classification (14) was found, with local vegetation and a well-circumscribed perforation defect in the right coronary cusp. Aortic valvuloplasty was performed involving reconstruction of the right coronary and non-coronary cusps using autologous pericardium, as well as abscess drainage and a vegectomy. The mitral annulus was dilated, with no valvular damage; thus, a suture annuloplasty from the anteromedial to the posterolateral commissure was performed. Finally, a fistula was noted between the left ventricle and the right atrium and was repaired by suture. The patient was progressively weaned from ECMO after 3 days, with good hemodynamic tolerance. Antibiotics were initiated after surgery using an association of ceftriaxone 100 mg/kg q.i.d., amikacin 7.5 mg/kg q.i.d., and vancomycin 15 mg/kg q.i.d.

Blood culture and PCR of the turbid pericardial fluid were both positive for *K. kingae*. Antibiotic treatment was thus adapted, with ceftriaxone 50 mg/kg q.i.d. alone for 4 weeks, as recommended by the American Heart Association (11). Postoperative recovery was characterized by a right hemiparesis with dysarthria. Brain MRI showed left frontal-parietal-insular ischemic lesions consistent with septic embolisms. The patient's neurological evolution was now satisfactory, with a progressive resolution of her symptoms.

**Figure 3:**
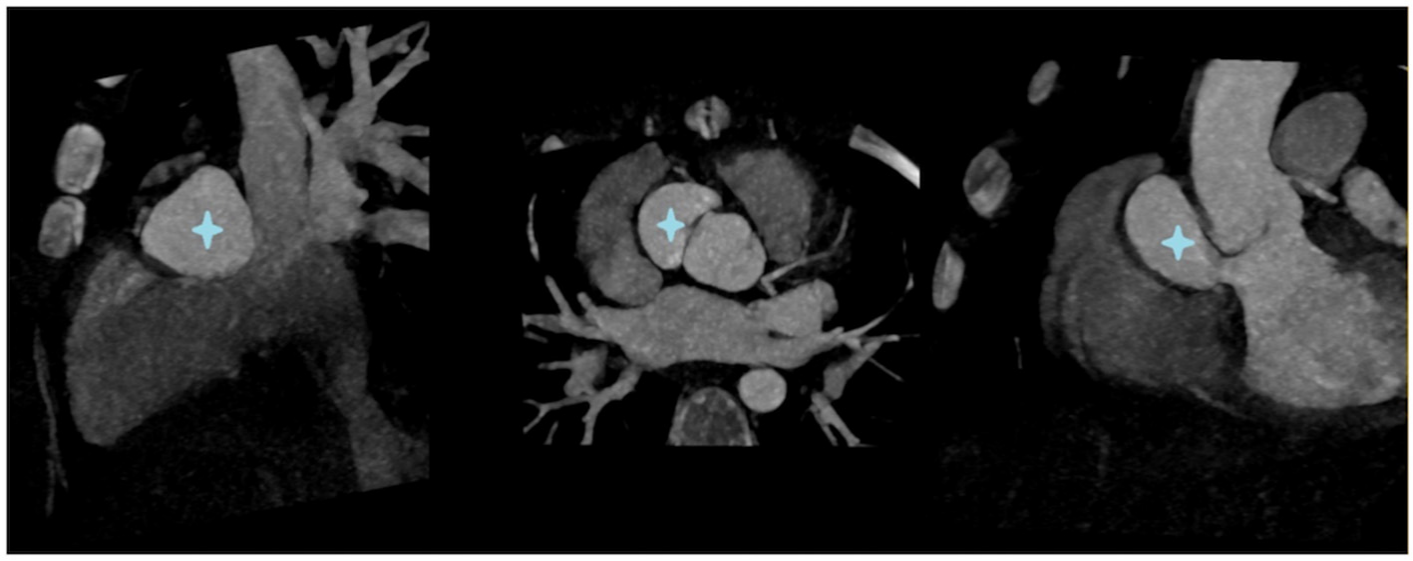
CT scan showing a para-aortic pseudoaneurysm (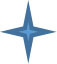).

However, after 3 months, she developed a large, para-aortic pseudoaneurysm with a moderate aortic regurgitation (Figure 3). She underwent a second cardiac surgery involving resection of the pseudoaneurysm and aortic valve repair consisting of direct closure of a small perforation in the right coronary cusp. The postoperative course was uneventful, and she was discharged after 5 days.

At the latest follow-up (3 years after IE), TTE was unremarkable except for a very mild residual aortic regurgitation. The girl is very active, with only a very mild residual sensory-motor hemiparesis.

**Figure 4:**
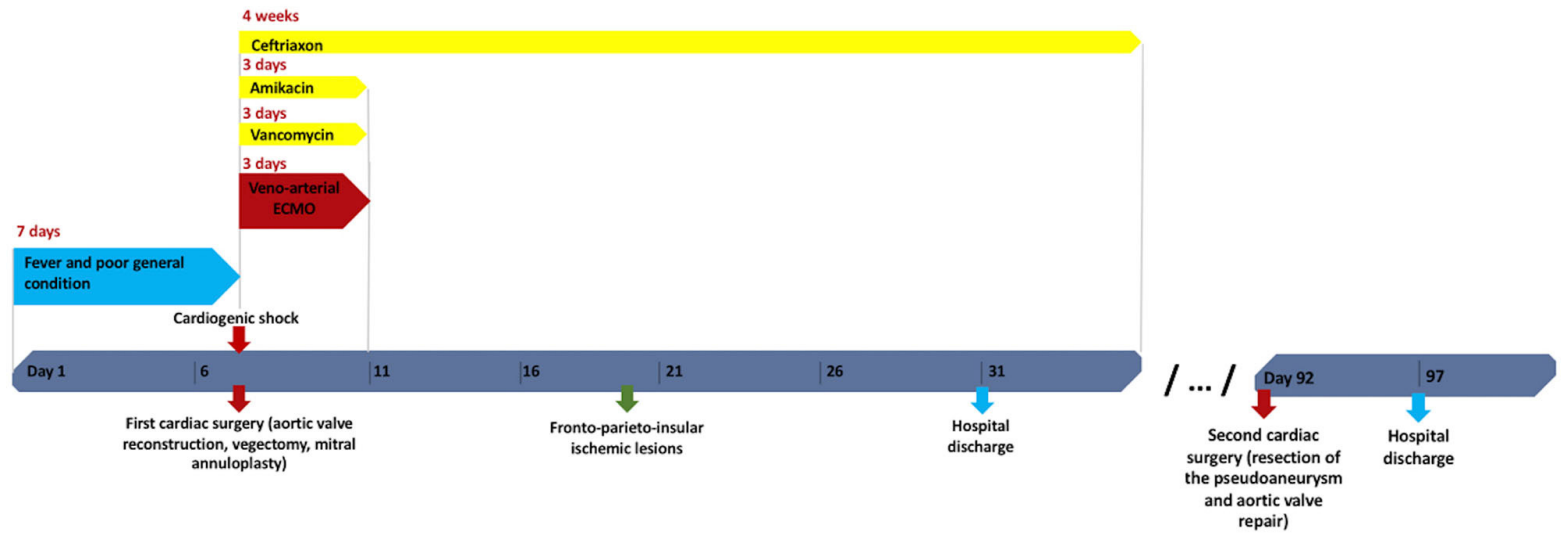
Timeline of main events. ECMO, extracorporeal membrane oxygenation.

The chronology of events is shown in Figure 4.

## Discussion

*K. kingae* belongs to the HACEK group of gram-negative bacilli that form a normal part of the human flora: *Haemophilus* species, *Aggregatibacter actinomycetemcomitans, Cardiobacterium hominis, Eikenella corrodens*, and *Kingella* species. All these gram-negative bacteria grow slowly, prefer a carbon dioxide-enriched atmosphere, and share an enhanced capacity to produce endocarditis, especially in children and young adults. Based on broad reviews, HACEK organisms are responsible for 3–10% of cases of native valve IE in children (15). Because of their fastidious, slow growth, they often cause culture-negative endocarditis, although modern culture and genomic identification techniques are now challenging this paradigm.

The present case highlights that IE is, without doubt, the most severe form of *K. kingae* infection, leading to severe life-threatening complications such as the destruction of heart valves, cardiac failure, septic shock, mycotic aneurysms, cerebrovascular complications, and embolic events. Many virulence factors have been identified in the pathophysiological mechanisms of invasive *K. kingae* infection. Kehl-Fie et al. showed that *K. kingae*'s pili enable it to adhere to the respiratory and synovial epithelium and probably to endocardial cells. They also described how piliation was higher among respiratory and non-endocarditis blood isolates and lower among invasive isolates. They found that piliation diminished progressively during the development of an invasive infection, and they concluded that pili might provide a selective advantage during the early stages of infection but a selective disadvantage during later stages (16). Furthermore, *K. kingae* is protected from phagocytosis by a polysaccharide capsule. Finally, the RTX toxin has been identified as a broad spectrum cytotoxic virulence factor, and it may lyse epithelial cells, synoviocytes, and macrophage cell lines (17).

This case was unusual because the patient presented with concomitant lesions to two valves. A review of the literature on *K. kingae* endocarditis among children younger than 16 years old identified 30 articles describing 43 patients. Thirty-seven (86.4%) of these patients were <4 years old. A specific valvular disease was mentioned for 36 patients, with only one sustaining lesions to two valves. The left side of the heart was more frequently involved, usually the mitral valve (91.9%). Valve lesion pathogenesis was considered to result from multiple causal factors, including a vegetation or a perforation of the valve. Indeed, perforation of the leaflet was reported in 18.9% of the affected valves, whereas a prior congenital disease was described in 31.8% of cases (12). A more recent publication comparing childhood *Kingella* and non-*Kingella* (caused by *staphylococci* and *streptococci)* IE in Israel described a *Kingella* group of 19 children with a mean age of 16 months old. Valvular impairment was reported in 58% of the children with *Kingella* IE, 37% suffered congestive heart failure, and 8% required urgent heart surgery. However, these numbers were similar in the non-*Kingella* IE group (13).

Our patient also sustained neurological complications that resolved during follow-up. Neurological events are the most common extracardiac complications of IE and are secondary to the migration of vegetation fragments (ischemic strokes, brain hemorrhage), infectious complications (brain abscess, meningitis), and non-specific complications (encephalopathy, convulsions, and headache). Of the 43 patients reported in the literature, 16 (36.4%) sustained serious neurological complications, and four died. Stroke was the most common neurological complication reported (>80%), surpassing meningitis or meningeal reaction, brain abscess, mycotic aneurysm, and intracranial hemorrhage (12). The results reported in the Israeli study were consistent with these data, with central nervous system complications occurring in 21% of children with *Kingella* IE (13). Three possible mechanisms are involved in the pathogenesis of stroke: cerebral infarcts secondary to septic emboli; rupture of a vessel wall affected by septic arteritis; or rupture of a mycotic aneurysm. An endothelial lesion is a prerequisite step in thrombus formation as it fosters the interaction between subendothelial connective tissue and circulating platelets and fibrin. As a continuation of the pathogenic process, an episode of superimposed bacteremia leads to the formation of a vegetation—a variably sized amorphous mass of platelets and fibrin with enmeshed microorganisms and inflammatory cells. A clinically apparent acute brain embolization is estimated to occur in 30% of children with a left-sided IE (12). However, in adult IE up to 80% of cerebral emboli were clinically silent, as shown by recent MRI studies (18, 19). Our patient matched these observations. Indeed, her IE was located on the left side of her heart and thus was probably responsible for the cerebral embolism.

Our patient had to be operated on as an extreme emergency because her rapid hemodynamic deterioration led to cardiac arrest. Notwithstanding this, there is currently no consensus on the optimal timing of surgery for IE due to *K. kingae*. Current recommendations for the surgical management of pediatric IE are mostly based on adult studies. The principal reasons for emergency surgery are heart failure, valve dysfunction, and embolic phenomena. Considering the published data on children, the American Heart Association does not recommend performing preventive urgery to prevent embolic events (11). On the other hand, following their experiences of *K. kingae* endocarditis in children, Lenoir et al. suggested immediate surgery, even for patients without apparent neurological complications, in order to prevent septic embolization. Cardiac surgery for patients with neurological complications was deferred since surgery itself can cause postoperative neurological deterioration such as secondary cerebral hemorrhage (20). As mentioned above, our patient presented with a cerebral embolism despite emergency surgery, but the 7-day delay between symptom onset and that surgery may have been related to her embolic phenomena.

Among adults with IE, it is recognized that avoiding valve replacement decreases hospital mortality and improves long-term survival (21). Valve preservation is also of paramount importance among children, whether their surgery is urgent or elective. Lower mortality rates and longer times free of reoperation have been reported in children who underwent native valve repair rather than a valve replacement (22). One of our main therapeutic aims and challenges was, therefore, to avoid valve replacement. The pseudoaneurysm can be a complication of surgery because of the frailty of valvular and perivalvular tissue; however, an infectious origin (mycotic aneurysm) cannot be excluded. The second surgery went well, with no complications, and the final result was very satisfactory: valvular function and growth capacity were preserved. It is important to note that the desire to preserve a valve should not delay surgery: valve replacement sometimes represents a life-saving procedure in the acute phases of an infection or, at a later time, a means of improving deteriorating cardiac function. More studies on larger populations are needed to determine the best surgical management timings and techniques.

## Data Availability Statement

The data analyzed in this study is subject to the following licenses/restrictions: The data are available in the patient's chart. Requests to access these datasets should be directed to raphael.joye@hcuge.ch.

## Ethics Statement

Written informed consent was obtained from the minor(s)' legal guardian/next of kin for the publication of any potentially identifiable images or data included in this article.

## Author Contributions

RJ and DC wrote the first draft of the manuscript. All authors contributed to conception of the article, manuscript revision, read, and approved the submitted version.

## Conflict of Interest

The authors declare that the research was conducted in the absence of any commercial or financial relationships that could be construed as a potential conflict of interest.
